# Smartbins: Using Intelligent Harvest Baskets to Estimate the Stages of Berry Harvesting

**DOI:** 10.3390/s19061361

**Published:** 2019-03-19

**Authors:** Patricio Galeas, Carlos Muñoz, Juan Huircan, Mario Fernandez, Luis A. Segura-Ponce, Cristian Duran-Faundez

**Affiliations:** 1 Department of Computer Science, Universidad de La Frontera, Temuco 4811230, Chile; 2 Department of Electrical Engineering, Universidad de La Frontera, Temuco 4811230, Chile; carlos.munoz@ufrontera.cl (C.M.); juan.huircan@ufrontera.cl (J.H.); 3 Department of Electrical Engineering, Universidad de Talca, Curicó 3344158, Chile; mafernandez@utalca.cl; 4 Department of Food Engineering, Universidad del Bío-Bío, Chillán 3810000, Chile; lsegura@ubiobio.cl; 5 Department of Electrical and Electronic Engineering, Universidad del Bío-Bío, Concepción 4051381, Chile; crduran@ubiobio.cl

**Keywords:** harvest, stages, sensors, monitoring, berries, Smartbin

## Abstract

In some important berry-producing countries, such as Chile, the fruit is harvested manually. The markets for these products are generally very distant, and any damage caused to the fruit during harvesting will be expressed in its shelf life. The first step to understanding the harvesting process is to identify what happens to the harvest baskets in each stage (picking, wait-full, transport-full, freezing tunnel, emptying and transport-empty), allowing variables that can affect the shelf life to be identified. This article proposes the use of Smartbins, intelligent harvest baskets with sensors to collect weight, temperature, and vibration data. Combined analysis of the variables collected, using machine learning algorithms, allows the system to estimate which stage the basket is at with an accuracy of 80%, and to assess whether the fruit has been exposed to situations that could affect its shelf life. Due to imbalance characteristics of the data collected, the best results were obtained in longer stages (picking and wait-full stages with 89% and 86% respectively).

## 1. Introduction

According to the United Nations Food and Agriculture Organization (FAO), the production, export, and price of berries (blueberries, cherries, cranberries, gooseberries, strawberries) have grown steadily over the last 20 years. Berry production reached approximately $1.8\times 10^{7}$ tons in 2016. The main producers were China, Turkey, Mexico, Spain, Egypt, Poland, Canada, Germany, Chile, and Italy, which together represented more than the 58% of world production. The price of these fruit has also increased steadily in recent years, with blueberries fetching the highest price per ton. Large producers such as China, Turkey, and Mexico export only a fraction of their production, whereas countries such as Spain and Chile export almost all of it.

In the case of Chile, blueberry production is mainly intended for distant markets (e.g., North America, Europe and Asia), so the fruit spends a considerable time in transit to its destination. There are producers in the south of the country who attribute 20% of fruit damage to the vibrations generated by the poor state of the roads from orchard to packing facility [1].

Furthermore, Chilean berries are generally harvested manually, which involves many workers. In December 2012 alone, the fruit export sector demanded more than 10 million man days [2], representing an important part of production costs. Harvest times are shortened, however, when the fruit is at optimal ripeness, and at times when external variables such as temperature and vibrations affect product quality as little as possible.

To control the quality of the berries, various solutions for tracking agricultural products from packing to their arrival at the target market (post-harvest) have been studied, based mainly on measuring humidity and temperature in the transport vehicles. These studies incorporate the use of sensors, global positioning system (GPS) technology to locate the vehicle, general packet radio service (GPRS) for communications between the vehicle and the monitoring station, and radio frequency identification (RFID) to identify the products [2,3]. Some of these technological solutions are commercially available, and their use helps to guarantee that the fruit is transported in suitable conditions and within the times committed to by the company. Moreover, there are studies that in addition to measuring humidity and temperature, incorporate the concept of “intelligent containers” [4,5], which reduce the amount of data sent to the monitoring station.

Although these solutions are useful tools for controlling the fruit during transport from packing to the final market, they do not cover the specific harvest process; this is recognized by experts as a highly important operation in agricultural production, since any insufficiency during this phase may cause the loss of an entire year’s work [6]. Indeed, studies such as [7] show that mechanical damage (bruising) to some fruit, due to bumps, vibrations, and overweight during the harvest, impacts directly on the shelf life of the product [6,8,9,10].

Patents have also been established for post-harvest solutions. The patent in [11] describes a system for monitoring and recording variables inside a fruit container, including geographical position, humidity, time, temperature, shock, vibration, ambient light and emissions of gases (carbon dioxide and ethylene). The system also includes sensors with wireless features (using technologies such as Bluetooth and Wi-Fi). The patent in [12] specifies a system for the packaging of oranges and tangerines with weight and ethylene adsorption sensors, including a data display module.

Other patents, as in Schueller et al. [13] and Xu et al. [14] propose products that can be used in orchards. The former [13] offers a solution for collecting and managing data during the manual processes of harvesting, planting, cutting flowers, pruning, and thinning of fruit trees. It includes an accelerometer, magnetic sensor, pedometer, RFID transponder, GPS, acoustic sensor, biosensors, and optical sensors to detect and analyze hand movements. This product is a sophisticated solution that needs to be customized to the specific crop. The second solution [14] consists of a Berry Impact Recording Device (BIRD), implemented as an artificial berry with an accelerometer and a microcontroller inside. This product is used to analyze the impact of mechanized picking processes [15,16] on berries; it measures vibrations in one artificial berry, which is insufficient information for harvest analysis.

To verify harvest conditions in situ, technologies based on wireless sensor networks (WSN) have been tested which incorporate low-cost, energy-saving technologies with acceptable computing capacities [7,17,18,19]. However, WSN must still be integrated with other communication systems, such as Wi-Fi or GSM/GPRS, so that important data reach the user in the shortest possible time. Ampatzidis et al. [20,21,22] address the issue of technology integration including RFID technology and bar codes in harvest bins and cherry orchards. This system, with the aid of an electronic scale and a differential global positioning system (DGPS) mounted on the tractors that transport the harvest bins, allows the weight of the harvested fruit to be measured and productivity indicators to be determined for orchards and pickers. Tan et al. [23] and Ampatzidis et al. [24] studied the integration of the above technologies with cloud-based monitoring systems, generating a harvest-tracking system in which information is available to local or remote users. These systems include visualization platforms to analyze and search for information, putting a decision-making tool at the user’s disposal. In addition, Ampatzidis et al. establish that weighing with an electronic scale on the tractor-trailer that transports the bins increases loading time by almost 33% [21]; this extra time could be eliminated if the weight could be measured in the harvest basket itself using an automatic weighing mechanism. It should be stressed that the studies cited do not consider environmental variables such as high temperatures and/or excessive vibrations to which the fruit is subjected, which, as mentioned previously, affect its shelf life.

**Figure 1:**
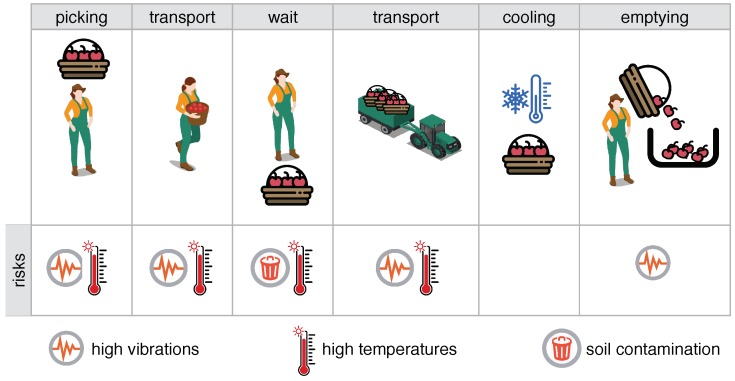
The harvest process and the risks that can affect the shelf life of the fruit.

To illustrate the aspects mentioned above, Figure 1 shows the blueberry harvest process in an orchard in southern Chile, where the harvest basket passes through several stages before arriving at the packing facility: picking, transport on foot, waiting times, transport by vehicle, refrigeration chain, emptying of the basket in the packing area. At each of these stages there are potential risks that could affect the final shelf life or some quality indicators. At least three important risks are identified: (1) excessive vibrations or bumps, (2) high temperatures and (3) contamination due to contact with the soil. Extreme vibrations due to rough handling or falling from the basket can occur during picking. The likelihood of vibrations is high during transfer on foot from plant to plant, transport by vehicle and, finally, the emptying stage. Exposure to direct sunlight or high temperature during extended periods of time could shorten the fruit’s shelf life; this risk occurs when the temperature in the harvested fruit is high during long periods, and it could be triggered during the stages of picking, waiting for transportation or transportation. During the harvest process, the picker needs to take occasional breaks, sometimes leaving the basket resting on the ground. According to agriculture regulations, the basket must not touch the ground due to possible soil (dirt) contamination of the fruit, so these events need to be avoided.

In view of the above, any improvement in the agricultural production process can have a significant impact, particularly in countries where this industry represents an important percentage of the productive matrix. It is also important for the berries business model that all production stages of harvesting should be monitored, so that potential damage to the berries can be evaluated. To do this, the first step is to implement a system capable of detecting and isolating each of these stages based on monitoring variables that can be measured during the whole process. This system should be simple and inexpensive; moreover, it should be modular and easily scalable to field size. Since it is necessary to monitor variables that affect the harvested fruit physically, the sensors should be placed as close to the fruit as possible. In this case, the inside of the harvest basket will be the ideal place to implement solution.

This article proposes a stage monitoring system for berry harvesting based on three variables measured with sensors inside the harvest basket: weight, temperature, and vibrations. Combined analysis of these variables can help to identify the different stages of the harvesting process and detect possible risks of excessive vibrations and high temperatures. This information also makes it possible to assess whether harvest operations are performed accurately.

The remainder of this paper is organized as follows: In Section 2 we present the proposal of this paper, describing the harvesting problem adopted as the case study and the proposed ‘Smartbin’ system, which includes modified harvest baskets fitted with various kinds of sensors, and a data acquisition module, allowing data collection during the different stages of the harvesting process. We also briefly describe the experimental design. Experimental results obtained by applying Smartbins in a real harvesting process are described in Section 3. The results show how the data collected from the instrumented baskets can serve to identify and describe the different stages of the harvesting process. Finally, Section 4 offers our conclusions and suggests some future directions for this work.

## 2. Materials and Methods

### 2.1. The Blueberry Harvesting Process

**Figure 2:**
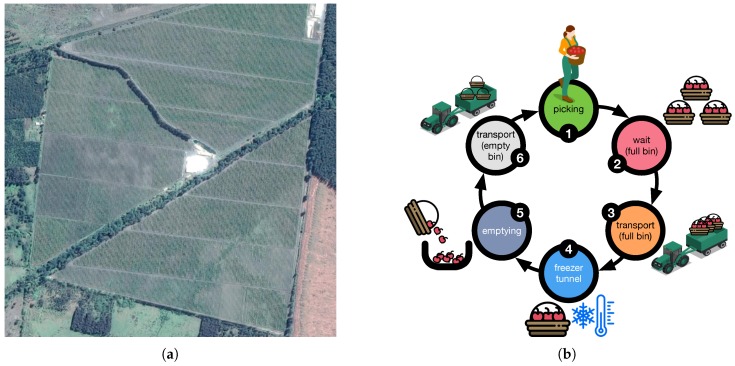
(**a**) Aerial view of the blueberry site owned by El Boldo, located in the municipality of Yungay in Chile’s VIII Region (Lat: −37.1149584, Long: −72.1973101), and (**b**) the six processing stages of a basket in blueberry harvesting at El Boldo.

As mentioned above, in Chile the complete production of blueberries is harvested manually, presenting interesting scenarios for testing the functionality of the prototype in real conditions. The experiments presented in this work were performed at El Boldo S.A., an agriculture company located in the municipality of Yungay, Ñuble Region, Chile, with 50 hectares of blueberries laid out in rows approximately 100 m long. As illustrated in Figure 2a, the packing facility is located at the center of the orchard; roads divide the blueberry plantation into 3 sectors, with each sector being divided into 7 sub-sectors for irrigation. Each sector has different varieties, including: Duke, Rabbiteye, Brightwell, Tifblue, O’Neal and Brigitta. The complete harvesting process is performed manually, beginning with the assignment of a group of pickers to each sector of the orchard. In Stage 1 (picking), the pickers, provided with a 3.5 L plastic basket hung around the neck by a harness, work down the rows. Picking takes 20 to 40 min per basket, depending on the picker’s experience and the volume of fruit on the shrubs. When the basket is full, the picker goes to the collection center (a shaded area), where the basket is received for counting (Stage 2: wait-full). The picker is provided with an empty basket to begin the process again; the full baskets remain at the collection center waiting for the tractor-trailer to take them to the local packing facility (Stage 3: transport-full). Prior to admittance to packing, the (full) baskets are placed on an electronic scale to weigh the fruit and a sample is taken to measure the temperature. The fruit is admitted to packing via a conveyor belt, where a cooling system lowers the temperature in a cold tunnel (Stage 4: Cold Tunnel). There is a company policy that says that the time from fruit picking to admittance to packing should not exceed 2 h. When a company does not have local packing, the baskets are transported in trucks to an export company, which is responsible for packing and shipping to the target market. These six stages, through which each harvest basket passes during blueberry harvesting at El Boldo, are illustrated in Figure 2b.

### 2.2. Proposal: The Smart Harvest Basket (Smartbin)

**Figure 3:**
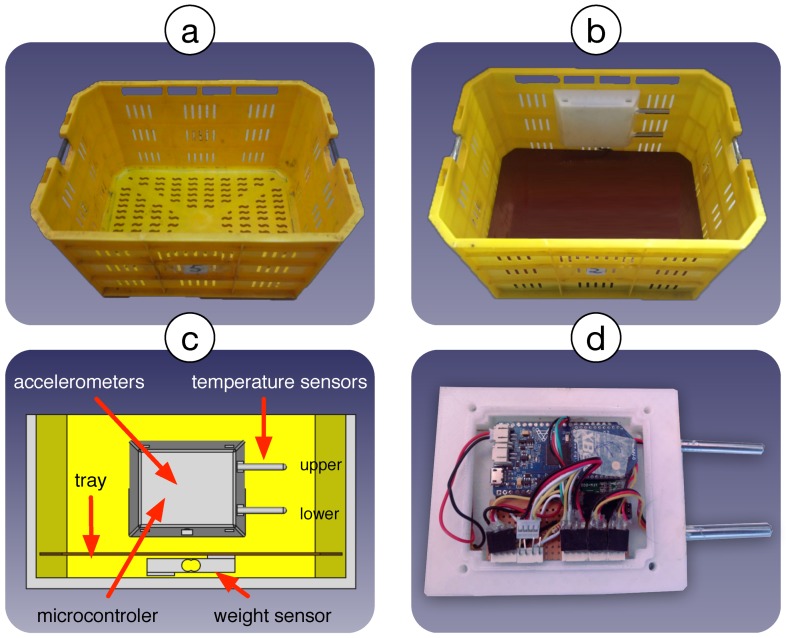
Different views of the 3.5 kg blueberry harvest basket. (**a**) Empty harvest basket. (**b**) Harvest basket with the monitoring system inside. (**c**) Vertical section of the basket with details of the housing of the electronic devices, temperature sensors (upper and lower), weighing tray, and weight sensor. (**d**) The internal electronics of the monitoring system.

The proposed monitoring system consists of incorporating vibration, weight, and temperature sensors into a berry harvest basket. To do this, a modular system was developed which can be easily installed inside the basket. The prototype is not commercially available and was constructed by the authors according to the following design requirements: the system must occupy a maximum 10% of the volume of the basket, it must not contain elements that can cause damage to the fruit, and the total weight of the system must not exceed 500 g. A 3.5 kg blueberry harvest basket used as a model is shown in Figure 3a. Figure 3b shows the monitoring module attached to the side and the weighing tray on the base, both inside the basket. Figure 3c illustrates the complete system, including the container with the electronic devices, the temperature sensors, the weighing tray and the load cell. We call this proposal ‘Smartbin’. Finally, Figure 3d shows the inside of the housing containing the prototype module.

The proposed module consists of two basic components: (1) the control module installed on one side of the basket, containing a microcontroller, a vibration sensor and temperature sensors, and (2) an electronic scale installed on the base of the basket, composed of a load cell (or weight sensor) and tray.

#### 2.2.1. The Data Acquisition Module

The data acquisition module consists of a SODAQ Autonomo (https://shop.sodaq.com/sodaq-autonomo.html) microcontroller, a real-time clock, two temperature sensors and an inertial measurement unit (IMU). The SODAQ Autonomo card uses a 32-bit Atmel SAMD21J18 processor, working at 48 MHz, with 256 kB of Flash memory and 32 kB of SRAM. It has a slot for a microSD card for internal data storage. A real-time clock (DS1307) is incorporated into the device and date and time information is associated with all captured data. The IMU is based on the MPU-9250 chip containing an accelerometer, gyroscope, and a 3-axis magnetometer. The system also has two temperature sensors based on the DS18B20 digital device, which provides an accuracy of 0.5 °C. As Figure 3 illustrates, these two sensors stick out as two tubes from the main device attached to one of the inner walls of the basket, allowing the temperature of the fruit to be measured at 6 cm and 10 cm from the base. The data acquisition module also works as a data logger, i.e., it stores the captured data on the microSD card for further processing. This card holds two types of records: the first corresponds to the IMU measurements, taken every 100 ms, and the second contains temperature and weight measurements collected every 15 s. A date-time stamp is associated with each record. Finally, the system was provided with a Li-Ion battery, 2300 mAh/3.7 V, with power to operate continuously for 30 h.

#### 2.2.2. The Electronic Scale

The electronic scale consists of a removable tray supported by a load cell to measure the fruit weight inside the basket. The weight system uses the YZC-133 load cell with a maximum load of 5 kg and an output of (1±0.15) mV/V. The load cell is connected to the HX711, a 24-bit analog-digital converter that sends the digitalized data to the SODAQ microcontroller through a serial communication interface. The electronic scale was tested in the laboratory to ensure a maximum measurement error of 100 g, which is considered sufficient for this application.

### 2.3. Experiment Design

To verify that the prototype works, five monitoring modules were built for testing under real harvest conditions on a blueberry plantation belonging to El Boldo S.A. The modules were installed in five harvest baskets (see Figure 3b) to facilitate monitoring of the harvest process based on the temperature, vibration, and weight records. Each Smartbin was assigned to a picker, who carried out the harvest process as usual so that the collected data would be representative of the actual process in real use conditions.

At the end of the working day, the experimental baskets were recovered and the SD cards with the data were extracted for analysis. During the experiment, we recorded some measurements manually to complement the data collected by the baskets, such as waiting times of the baskets, times of exposure to the sun with picked fruit, times in packing area, times in harvesting, etc.

## 3. Results

**Figure 4:**
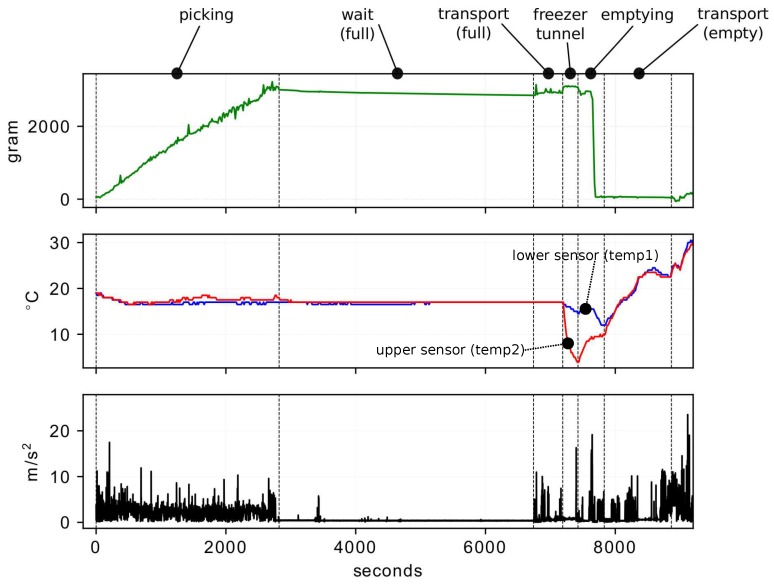
Summary of results of vibrations, weight, lower temperature (temp1) and upper temperature (temp2) sensors, for the first picking of the harvest basket corresponding to Node 1.

The data obtained from the five baskets presented similar behavior. Figure 4 depicts the vibrations, weight, and temperature data obtained for one harvest basket.

The weight graph illustrates the mass of the fruit contained in the basket in grams. The temperature graph is measured in degrees Celsius and represents the temperature inside the basket at both the lower sensor (temp1), and the upper sensor (temp2). Finally, the graph of the vibrations (accelerations) is represented with the module of the vector sum of the linear accelerations on the three spatial axes and given by Equation (1) and expressed in $m/s^{2}$.

(1) $$A_{T}=\sqrt{acc_{x}^{2}+acc_{y}^{2}+acc_{z}^{2}}$$

All the recorded variables were aligned on the same time scale (seconds), where zero corresponds to the point at which the monitoring system in the basket was switched on. The vertical dotted lines correspond to the six harvest stages mentioned in Figure 2b, which were manually determined with the information compiled during the experiment. Next, the behavior of the three variables monitored for each of these stages is described:

1. **Picking**: The records of the accelerometer clearly indicate the movements of the basket during harvesting. The weight sensor shows a progressive increase as the basket fills. The temperature sensors record values similar to the environmental temperature.
2. **Wait (Full)**: During this period, the accelerometer does not register significant movements. The weight sensor does not register variations in mass in the basket. The temperature sensors continue to record the environmental temperature.
3. **Transport (Full)**: Here the accelerometer again detects movements of an order similar to that of picking; these movements are also detected by the weight sensor due to the vertical accelerations associated with transport by vehicle. The temperature sensors continue to record the environmental temperature.
4. **Cold Tunnel**: In this process the full basket moves through a cold tunnel on a low-speed conveyor belt. Here, the vibrations detected are imperceptible at the scale of the graph. The weight sensor also does not record changes in mass inside the basket. The two temperature sensors record a significant loss, with the higher sensor in the basket recording the greatest temperature change due to the characteristics of the cooling system.
5. **Emptying**: Emptying the fruit onto a conveyor belt generates significant vibrations detected by the accelerometer due to tipping of the basket. At this point the weight sensor drops drastically due to the loss of mass inside the basket. Given that this process occurs outside the cold tunnel, the temperatures of both thermometers begin to rise significantly.
6. **Transport (Empty)**: The vibrations from transporting the empty basket are clearly recorded by the accelerometer. The weight sensor continues to record the absence of fruit, and the temperature sensors begin to register the increase in environmental temperature.

**Figure 5:**
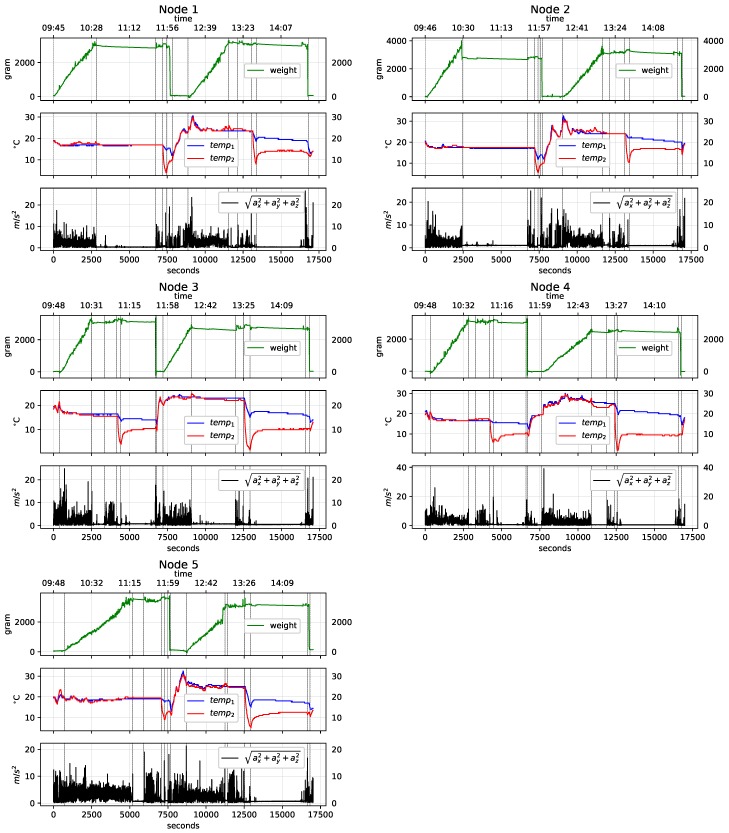
Visualization of the measures from the five monitoring nodes. Here the temperature, weight and vibration variables are observed during the harvest process synchronized on the time scale. The variables $temp_{1}$ (blue) and $temp_{2}$ (red) represent the lower and upper temperature in the harvest basket, respectively.

Figure 5 shows the vibration, weight and temperature graphs for the five nodes ($N_{1},N_{2},\ldots ,N_{5}$) used in the experiment, for an entire working day. During the working day, each node described two picking processes (harvest 1 and harvest 2), yielding a total of 10 recorded harvests. The graphs also contain vertical lines that define the times in seconds at the beginning and end of each stage of the harvest process. The first harvest starts at second 0 (typically at 9:45 a.m., depending on picker assignment), so the temperature is low (no more than 18 °C). The second harvest begins around second 8000–9000 (midday), when the temperature rises to 25 °C–30 °C depending on the weather conditions.

**Figure 6:**
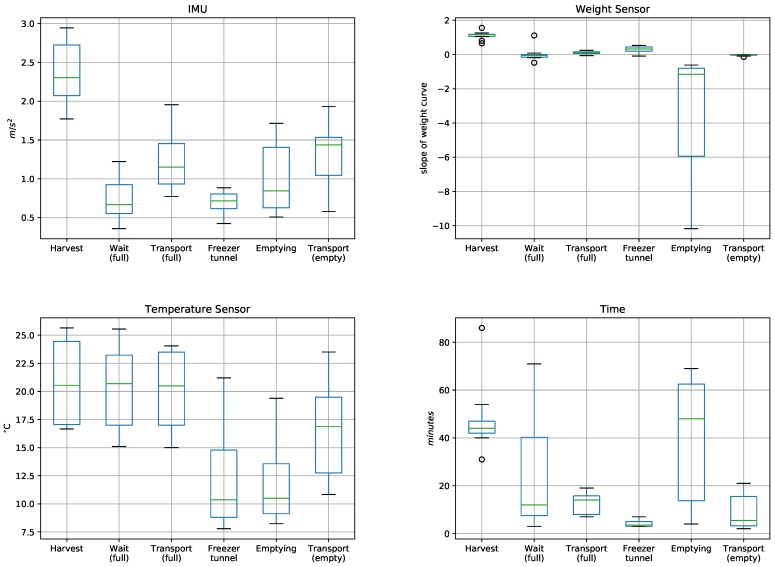
Boxplot of the results of the five nodes for each stage of the harvest process.

Figure 6 presents a statistical analysis that integrates the data from the 10 harvests, grouped by the results from each sensor (IMU, weight sensor, temperature sensor) and the times associated with each stage. In the case of the weight sensor, an indicator of the variation in weight over time was calculated (grams/second), which corresponds to the slope of the weight curve. Please note that this graph shows values that can differentiate some of the harvest stages. For example, in the IMU the mean values for picking and transport exceed the other stages, reaching values over 1. In the case of the weight sensor, the slope of the curve during picking stands out, maintaining values near 1, whereas during emptying a negative slope is produced due to the steep weight loss. The temperature sensor yields significantly lower values than the environmental temperature only during the stages of cold tunnel and emptying.

When the duration of each stage is analyzed, long times are observed for picking, wait-full, and emptying. During the first and second stages, the fruit is subjected to temperatures that could affect its shelf life due to dehydration. In the emptying stage, the fruit must wait for classification into different qualities.

### 3.1. Algorithms for the Recognition of Harvest Stages

To generate a recognition mechanism for the different stages of the harvest based on the variables monitored, different classification strategies were analyzed based on machine learning (ML) algorithms. To do this, we used the programming language Python and five libraries: scipy, numpy, matplotlib, pandas, and sklearn.

**Figure 7:**
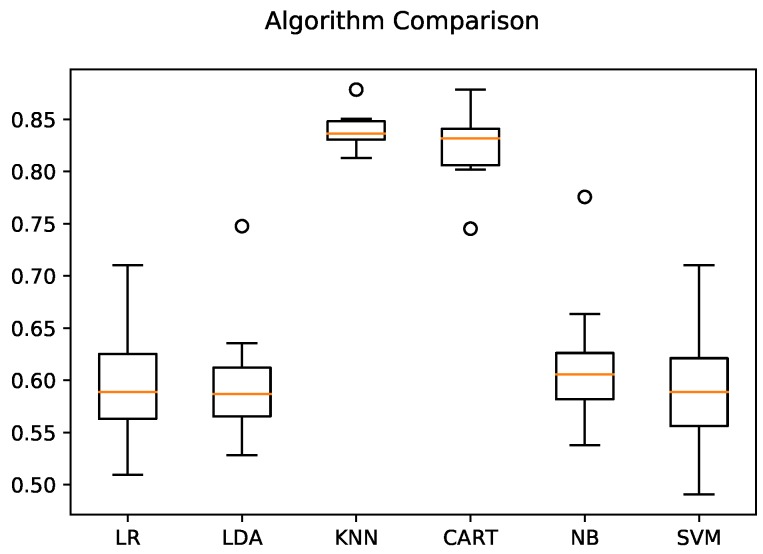
Boxplot of comparison of the ML algorithms on the harvest data. The KNN and CART algorithms stand out clearly from the rest.

The data from the 10 harvests were pre-processed and tabulated in a csv text file of 1669 records in 8 columns: *base_time*, *id_device*, *imu_mean*, *weight_slope*, *weight_mean*, *temperature_mean*, *temperature_slope*, and *state*. The mean values were calculated using an arbitrary interval of Δt=50 s. In the tests, six ML algorithms were evaluated: logistic regression (LR), linear discriminant analysis (LDA), K-nearest neighbors (KNN), classification and regression trees (CART), Gaussian Naive Bayes (Gaussian NB) and support vector machines (SVM). We used the data from four of the five nodes (equivalent to eight harvests), while the data from Node 3 (equivalent to two harvests) was left for analysis to provide an independent final validation of the selection process of the recognition model. To obtain the optimal model, k-fold cross-validation was used with 80% of the date to generate the model and 20% to validate it. Figure 7 shows a comparison of the ML algorithms mentioned above; the KNN and CART algorithms stand out, presenting accuracies of over 80%.

**Figure 8:**
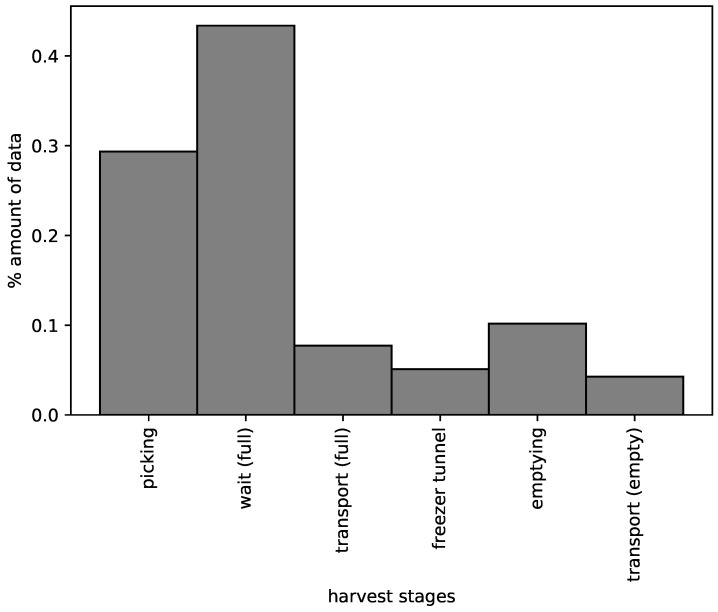
Histogram representing the amount of data available (observations) for each harvest stage. Here we observe the typical case of imbalanced classes.

Table 1 shows the result of the KNN classifier and the validation data set. Here, we observe that all stages present relatively high performance values except the freezer tunnel and transport (empty) stages. On analyzing the records, we find that only a low amount of data (10%) corresponds to these two harvest stages, while about 90% of the observations correspond to the other stages. This situation is evident in Figure 8, where we find the typical case of skewed classes or a class imbalance problem [25]. The low performance could be explained because the KNN algorithm does not classify the harvest stages well with the proportionally small amount of data available. Table 1 also includes two support values for each stage: the standard support, and the support in seconds. The latter represents the amount of time the basket remains in each stage. The basket remains a long time in the stages picking and wait (full), and for these stages the precision and recall values are high, meaning that the time estimation is reasonable. On the other hand, in the transport (full), freezer tunnel and emptying stages, the precision and recall values are lower, probably because these stages are short causing the problem of imbalanced data.

### 3.2. Validation of the Model with an Independent Node

**Figure 9:**
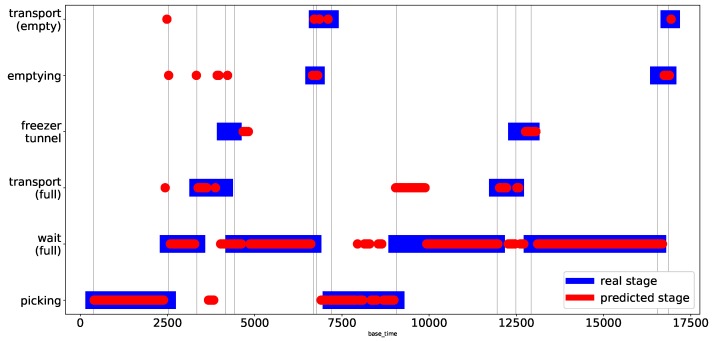
This graph shows a comparison between the stages estimated by the KNN classifier (red circles) and the real stages of the harvest associated with Node 3 (blue rectangles).

Finally, we decided to execute one last validation process of the KNN model with data from Picking Node 3. This guarantees total independence of the validation, since none of the data produced by this physical node were used to generate the recognition model. Table 2 shows the precision of the model based on this independent node. Here, a slight drop in performance is noted (from 82% to 79%), as well as a variation in the individual performance. This result confirms that the KNN algorithm performs acceptably in harvest stage recognition Finally, Figure 9 shows a comparison between the values of the harvest stages estimated by the KNN model (red line) and the real harvest stages of Node 3 during the field test (blue line). The red circles on the blue lines correspond to the values successfully estimated by the model, corresponding to approximately 80% of cases.

## 4. Discussion

In many countries, berry picking is still done manually. This makes it difficult to control the harvest process, which can influence the ultimate quality of the harvested fruit due to human and/or environmental factors. Although many of the variables associated with the harvest process (vibrations, temperature, overweight, etc.) are generally difficult to control, they can be measured using embedded sensor systems that can be incorporated into the fruit harvest baskets.

Using the Smartbin in a real harvest situation, we gathered temperature, weight and vibration data from which to construct a mathematical model that can identify at which stage of the process each Smartbin is. The model was constructed comparing six different ML techniques (LR, LDA, KNN, CART, Gaussian NB, and SVM). The best performance was obtained with KNN (close to 80%). Due to the imbalanced nature of the data collected, the best results were obtained in longer stages (picking and wait-full, with a performance of 89% and 86% respectively); while in shorter stages, representing about 10% of the total amount of data, the performance decreased considerably (to between 36% and 60%).

Two similar solutions were found in the literature. Xu [14] uses an artificial berry to analyze vibrations during the harvest process. It is a simple approach based on an accelerometer, but insufficient to generate enough information to identify harvest stages. Schueller’s patent [13] consists of a generic platform customizable for different agricultural processes; it includes sophisticated sensors and algorithms to recognize manual agricultural operations (events). The Smartbin presented here generates important information for the producer, including the number of baskets at each harvest stage, process bottlenecks, excessive times when the fruit is subjected to undesirable conditions (temperature and vibrations), estimation of production time intervals, identification of workers who mishandle the basket, etc.

The cost of the monitoring system is €103; this value was calculated using the price of each component of the prototype and does not include the price of the harvest basket.

Despite the low complexity of the prototype, it is estimated that its design can still be improved by eliminating the mechanical component used to measure the mass inside the basket. This improvement would involve a simplification of system installation and maintenance, as well as a reduction in the power consumption and the volume occupied in the basket. To do this, it will be necessary to assess the feasibility of estimating the mass of the fruit indirectly based on a detailed analysis of the data generated by the IMU in conjunction with estimation models that include information about harvest sequence patterns.

In the future, our intention is to construct a model that can use factor analysis to estimate the shelf life of the fruit after exposure to extreme conditions of the variables measured. This system could form the basis of a decision support system (DSS) for berry harvesting, where the producer would know what to do with the different qualities of harvested fruit. For example, if the fruit is not damaged, it can be exported; otherwise, it can be sent directly to individual quick freezing (IQF), thereby avoiding future economic losses. On the other hand, the DSS could have real-time alarm systems to avoid the fruit being exposed to extreme environmental variables or mistreatment. Future efforts will also consider improvements in the classification algorithm by solving the imbalanced data problem. This can be achieved by increasing the amount of data in short harvest stages by simply increasing the sampling frequency of the sensors (temperature and weight). The monitoring systems will also address the incorporation of other kinds of sensors (such as positioning systems) and wireless communications, to offer online remote data access.

## Figures and Tables

**Table 1:** Performance of the K-Nearest Neighbors algorithm (KNN) using validation data.

STAGE	Precision	Recall	f1-Score	Support (Samples)	Support (s)
**picking**	0.90	0.90	0.90	87	4350
**wait (full)**	0.84	0.94	0.89	103	5150
**transport (full)**	0.75	0.39	0.51	23	1150
**freezer tunnel**	0.40	0.36	0.38	11	550
**emptying**	0.76	0.90	0.82	31	1550
**transport (empty)**	0.83	0.42	0.56	12	600
avg/total	0.82	0.83	0.82	267	13,350

**Table 2:** Performance of the KNN algorithm using the data from the independent node.

STAGE	Precision	Recall	f1-Score	Support (Samples)	Support (s)
**picking**	0.89	0.86	0.87	81	4050
**wait (full)**	0.86	0.86	0.86	194	9700
**transport (full)**	0.36	0.44	0.40	27	1350
**freezer tunnel**	0.36	0.29	0.32	14	700
**emptying**	0.33	0.50	0.40	8	400
**transport (empty)**	0.60	0.30	0.40	10	500
avg/total	0.79	0.78	0.78	334	16,700

## Supplementary Materials

Supplementary File 1

## References

[1] Rojas P. (2016). Cosecha de Arándanos Cierra Con Bajas Cifras Por Factores Climáticos; El Asustral de Osorno. https://sago.cl/cosecha-arandanos-cierra-bajas-cifras-factores-climaticos/.

[2] Oficina de Estudios y Politicas Agrarias (2012). Estimación y Caracterización de la Demanda de la Mano de Obra Asociada a la Fruticultura de Exportación.

[3] Santa J., Zamora-Izquierdo M.A., Jara A.J., Gómez-Skarmeta A.F. (2012). Telematic platform for integral management of agricultural/perishable goods in terrestrial logistics. Comput. Electron. Agric..

[4] Lang W., Jedermann R., Mrugala D., Jabbari A., Krieg-Brückner B., Schill K. (2011). The Intelligent Container: A Cognitive Sensor Network for Transport Management. IEEE Sens. J..

[5] Hidalgo E., García Oya J.R., Muñoz Chavero F., González Carvajal R. (2018). Intelligent Containers Based on a Low-Power Sensor Network and a Non-Invasive Acquisition System for Management and Tracking of Goods. IEEE Trans. Intell. Transp. Syst..

[6] Brasil I.M., Siddiqui M.W., Siddiqui M.W. (2018). Chapter 1—Postharvest Quality of Fruits and Vegetables: An Overview. Preharvest Modulation of Postharvest Fruit and Vegetable Quality.

[7] Saeed A., Reza G.H., Mohammad M., Hossein G. (2015). Mechanical Damage of Strawberry During Harvest and Postharvest Operations. Acta Tech. Agric..

[8] Jarimopas B., Singh S.P., Saengnil W. (2005). Measurement and analysis of truck transport vibration levels and damage to packaged tangerines during transit. Packag. Technol. Sci..

[9] La Scalia G., Aiello G., Miceli A., Nasca A., Alfonzo A., Settanni L. (2016). Effect of Vibration on the Quality of Strawberry Fruits Caused by Simulated Transport. J. Food Process Eng..

[10] Jarimopas B., Singh S.P., Sayasoonthorn S., Singh J. (2007). Comparison of package cushioning materials to protect post-harvest impact damage to apples. Packag. Technol. Sci..

[11] Weyna P.V., Fleming M.N. (2018). A Method and System for Monitoring Cargo Conditions. US Patent.

[12] Chen J., Yuan M., Peng C., Liao G., Li X. (2016). Oranges and Tangerines Retainfreshness Packaging Case That Can Weigh. CN Patent.

[13] Schueller J.K., Lange A.F., Rosa U.A. (2015). Intelligent Tool for Collecting and Managing Data during Manual Harvesting of Fruits and Vegetables. US Patents.

[14] Xu R., Li C. (2015). Berry Impact Recording Device. US Patent.

[15] Yu P., Li C., Rains G., Hamrita T. (2011). Development of the Berry Impact Recording Device sensing system: Hardware design and calibration. Comput. Electron. Agric..

[16] Yu P., Li C., Rains G., Hamrita T. (2011). Development of the Berry Impact Recording Device sensing system: Software. Comput. Electron. Agric..

[17] Yu X., Wu P., Han W., Zhang Z. (2013). A survey on wireless sensor network infrastructure for agriculture. Comput. Stand. Interfaces.

[18] Baronti P., Pillai P., Chook V.W.C., Chessa S., Gotta A., Hu Y.F. (2007). Wireless sensor networks: A survey on the state of the art and the 802.15.4 and ZigBee standards. Comput. Commun..

[19] Yick J., Mukherjee B., Ghosal D. (2008). Wireless Sensor Network Survey. Comput. Netw..

[20] Ampatzidis Y.G., Vougioukas S.G., Bochtis D.D., Tsatsarelis C.A. (2009). A yield mapping system for hand-harvested fruits based on RFID and GPS location technologies: Field testing. Precis. Agric..

[21] Ampatzidis Y.G., Vougioukas S.G. (2009). Field experiments for evaluating the incorporation of RFID and barcode registration and digital weighing technologies in manual fruit harvesting. Comput. Electron. Agric..

[22] Ampatzidis Y.G., Whiting M.D., Liu B., Scharf P.A., Pierce F.J. (2013). Portable weighing system for monitoring picker efficiency during manual harvest of sweet cherry. Precis. Agric..

[23] Tan L., Wortman R. Cloud-Based Monitoring and Analysis of Yield Efficiency in Precision Farming. Proceedings of the 2014 IEEE 15th International Conference on Information Reuse and Integration (IEEE IRI 2014).

[24] Ampatzidis Y., Tan L., Haley R., Whiting M.D. (2016). Cloud-based harvest management information system for hand-harvested specialty crops. Comput. Electron. Agric..

[25] Guo X., Yin Y., Dong C., Yang G., Zhou G. On the Class Imbalance Problem. Proceedings of the 2008 Fourth International Conference on Natural Computation.

